# Rv0132c of *Mycobacterium tuberculosis* Encodes a Coenzyme F_420_-Dependent Hydroxymycolic Acid Dehydrogenase

**DOI:** 10.1371/journal.pone.0081985

**Published:** 2013-12-11

**Authors:** Endang Purwantini, Biswarup Mukhopadhyay

**Affiliations:** 1 Department of Biochemistry, Virginia Tech, Blacksburg, Virginia, United States of America; 2 Virginia Bioinformatics Institute, Virginia Tech, Blacksburg, Virginia, United States of America; 3 Departments of Biological Sciences, Virginia Tech, Blacksburg, Virginia, United States of America; 4 Virginia Tech Carilion School of Medicine, Virginia Tech, Blacksburg, Virginia, United States of America; Concordia University Wisconsin, United States of America

## Abstract

The ability of *Mycobacterium tuberculosis* to manipulate and evade human immune system is in part due to its extraordinarily complex cell wall. One of the key components of this cell wall is a family of lipids called mycolic acids. Oxygenation of mycolic acids generating methoxy- and ketomycolic acids enhances the pathogenic attributes of *M. tuberculosis*. Thus, the respective enzymes are of interest in the research on mycobacteria. The generation of methoxy- and ketomycolic acids proceeds through intermediary formation of hydroxymycolic acids. While the methyl transferase that generates methoxymycolic acids from hydroxymycolic acids is known, hydroxymycolic acids dehydrogenase that oxidizes hydroxymycolic acids to ketomycolic acids has been elusive. We found that hydroxymycolic acid dehydrogenase is encoded by the *rv0132c* gene and the enzyme utilizes F_420_, a deazaflavin coenzyme, as electron carrier, and accordingly we called it F_420_-dependent hydroxymycolic acid dehydrogenase. This is the first report on the involvement of F_420_ in the synthesis of a mycobacterial cell envelope. Also, F_420_-dependent hydroxymycolic acid dehydrogenase was inhibited by PA-824, and therefore, it is a previously unknown target for this new tuberculosis drug.

## Introduction

**Figure 1:**
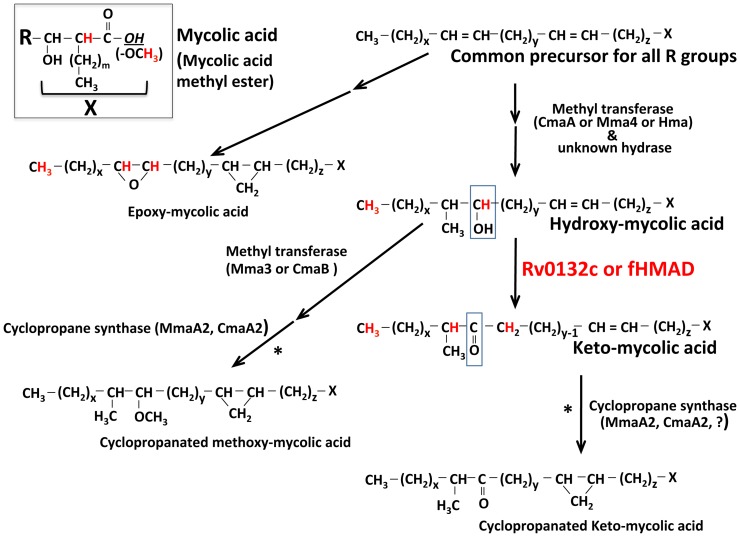
Proposed pathways for the synthesis of hydroxy-, keto-, methoxy- and epoxymycolic acids in mycobacteria [7], [24]. A common intermediate for various R groups is used as the starting point. Where MmaA2 and CmaA2 are involved in the formation of *cis* cyclopropane group, CmaA2 and an yet to identified enzyme (indicated by?) catalyze trans-cyclopropanation [48], [67]. The details of the individual R groups are shown in Fig. S1. * indicates that it is not known whether the cyclopropanation step follows or precedes oxygenation. All protons (except for the isolated groups) that have been target for NMR data analysis have been shown in red. The OH group shown in italics and underlined in the box at the left corner of the figure was converted to a methoxy group during saponification of mycolic acids; the process generated mycolic acids methyl esters (MAMEs).

The cell wall of *Mycobacterium tuberculosis* (*Mtb*), the causative agent of tuberculosis [1], [2], has an extraordinarily complex and very hydrophobic structure. Consequently it offers an exceptionally low permeability and makes the *Mtb* cells poorly accessible to drugs and less vulnerable to attack by the host immune system [3]. For this reason, cell wall synthesis enzymes of *Mtb* have been targeted for TB drug development [4]. Mycolic acids (MAs) are some of the key lipid components of the mycobacterial call wall. These “high-molecular weight beta-hydroxy fatty acids with a long alpha-alkyl side chain” [5] (Fig. S1) are constituents of mycolyl-arabinogalactan-peptidoglycan complex and trehalose mono-/di-mycolates (TMM and TDM) [6]–[8]. By helping to build a strong cell wall and being immunogenic [7], [9], [10], these complexes contribute to the development of TB [3], [10]–[15]. *Mtb* generates three structural types of MAs which are called α-, methoxy- and keto-mycolic acids (α-, M- and K-MAs) and under *in vitro* growth conditions it does not contain epoxymycolic acids (E-MAs) that are found in *Mycobacterium smegmatis* [16]; the respective chemical structures are shown in the Supporting Material (Fig. S1). The keto- and methoxy-derivatives enhance the pathogenic nature of *Mtb* [17], [18], and the bacterium uses these compounds to modulate the host immune response [9], [19]–[21]. A recent report shows that K-MAs allow *Mtb* to form pellicle structures, which in turn make this pathogen drug-resistant [22]. Thus, the enzymes that introduce keto- and methoxy-groups in mycolic acids are of research interest [3], [17], [23]–[26]. These oxygenated lipids are generated through common immediate precursors, hydroxymycolic acids (H-MAs) (Fig. 1) [3], [24], [27]. Whereas it is known that in *Mtb* the conversion of H-MAs to M-MAs is catalyzed by an adenosylmethionine-dependent methyltransferase (Mma3 or CmaB) encoded by the ORF Rv0643c [7], [24], [26] (Fig. 1), the enzyme that oxidizes H-MAs to K-MAs remains unknown. We call this unknown enzyme hydroxymycolic acid dehydrogenase (HMAD). In this report we describe the gene that encodes HMAD in *Mtb* and demonstrate that the enzyme utilizes coenzyme F_420_, a deazaflavin derivative, as electron carrier (Fig. 1). Thus, we named the enzyme fHMAD for F_420_-dependent Hydroxy Mycolic Acid Dehydrogenase. Also, we show that fHMAD is inhibited by PA-824, a nitroimidazopyran and a new TB drug that is currently on clinical trial [28].

## Results and Discussion

### Identification of Rv0132c as Coenzyme F_420_-dependent Hydroxymycolic Acids Dehydrogenase (fHMAD) in *M. tuberculosis*

This work began with an analysis of the available data, and the resulting hypothesis was tested via genetic analysis of an *Mtb* gene in *Mycobacterium smegmatis*. The rationale for the selection of *M. smegmatis* as the experimental host has been elaborated below.

#### Selection of Mycobacterium smegmatis as a facile screening host in a search for the HMAD encoding gene of Mycobacterium tuberculosis

As mentioned above, *Mtb* produces α-, K- and M-MAs, and it does not contain epoxymycolic acids (E-MAs) under *in vitro* growth conditions [16]. In this regard *Mycobacterium bovis* strain BCG (BCG) is similar to *Mtb* except some of the strains of the former do not produce M-MAs as the *cmaB* or *mma3* gene of the organism is non-functional due to a point mutation [16], [25], [26]. *M. smegmatis* produces α-, α′-, and E-MAs but is devoid of K- and M-MAs [16], [29]. The structures of these species are shown in Fig. S1; in *M. smegmatis* five variations of the α group, α1-, α2-, α3-, α4- and α5, are found [29]. The investigation described in this report concerns only the longer aliphatic chains (the R groups) of the MAs (Fig. S1 and Fig. 1).

Fig. 1 shows the proposed pathways for synthesis of H-, K-, M- and E-MAs in wild-type and recombiant *Mtb*, BCG and *M. smegmatis* [17], [23], [24], [27]; a common precursor for the aliphatic chains of various MAs serves as the starting point in this scheme. The deletion of the *hma* gene (also called *mma4* and *cmaA*) in *Mtb* abolishes the production of K- and M-MAs and causes the production of E-MAs and an intermediate that is similar to α-MAs of *M. smegmatis* [17], [23], [27]. Heterologous expression of the *Mtb* or BCG *hma* gene (*orf rv0642* or *mb0661*, respectively) in *M. smegmatis* allows the synthesis of H-MAs and reduces the production of α- and E-MAs in the recombinant strain [23], [24], [27]. Therefore, in *M. tuberculosis* the *hma* gene encodes the enzyme that generates H-MAs as precursors for both keto and methoxy forms, and this process competes well with the E-MA formation. The accumulation of H-MAs in a *M. smegmatis* strain carrying heterologous *hma* shows that the organism lacks both Mma3 (or CmaB) and HMAD and therefore cannot transform this intermediate into M-MAs and K-MAs [23], [24], [27]. Accordingly, a recombinant *M. smegmatis* strain carrying *Mtb hma* could be used to screen candidate *Mtb* genes for HMAD activity via complementation. This is advantageous, as unlike *Mtb, M. smegmatis* is not pathogenic and it grows much faster than *Mtb* or BCG [30].

#### Identification of rv0132c as a candidate gene encoding HMAD

**Figure 2:**
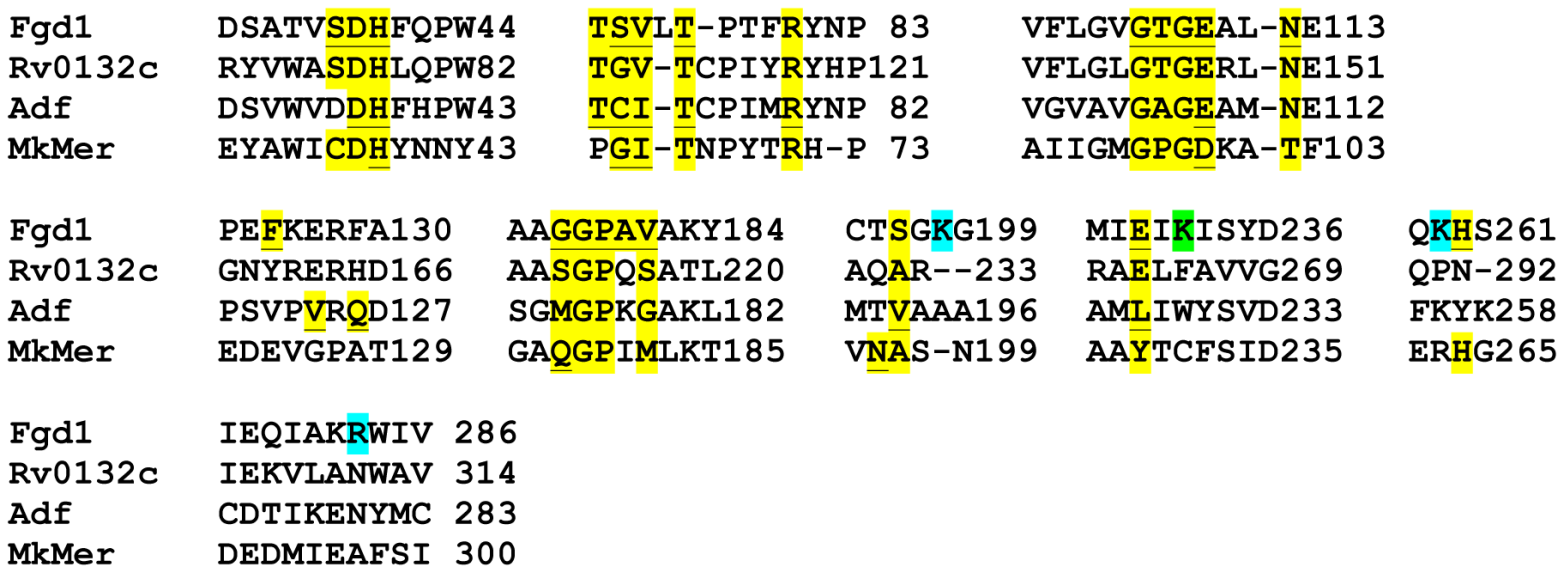
Comparison of the primary structures of Rv0132c (fHMAD) and three Mer homologs, Fgd1, MkMer and Adf. A ClustalW comparison was refined manually based on X-ray crystallographic structures of F_420_-dependent glucose-6-phosphate dehydrogenase (Fgd1) of *Mycobacterium tuberculosis*, F_420_-dependent methylenetetrahydromethanopterin reductase from *Methanopyrus kandleri* (MkMer), and F_420_-dependent secondary alcohol dehydrogenase (Adf) from *Methanoculleus thermophilicus* [32], [39], [40], [68]. Residue labels: yellow shade and underlined, determined F_420_-binding residue; yellow shade, predicted F_420_-intercating residue; turquoise shade, forming positively charged pocket for binding the phosphate of glucose-6-phopsphate in Fgd1; green shade, residue involved in binding a citrate ion [32].

We searched for this gene in the *Mtb* H37Rv genome [31] by using the following criteria. It must be present in both *Mtb* and BCG while absent in *M. smegmatis*. It should encode a dehydrogenase capable of catalyzing a two-electron transfer process, as the conversion of H-MAs to K-MAs involves the oxidation of a secondary alcohol group to a keto group. This dehydrogenase must also possess the structural elements for interaction with a hydrophobic substrate such as a mycolic acid. One of the *Mtb* ORFs that matched these characteristics was Rv0132c and it has been known as Fgd2 [31], [32]. It is a structural homolog of coenzyme F_420_-dependent glucose-6-phosphate (G6P) dehydrogenase (Fgd or Fgd1) that catalyzes two-electron oxidation of G6P [glucose-6-phosphate +F_420_ → 6-phosphogluconolactone + reduced F_420_ (F_420_H_2_)] [33], [34]. Coenzyme F_420_ is a deazaflavin derivative that is found in all mycobacteria [35], [36]. At the ground state it functions similar to nicotinamide coenzymes or NAD(P), mediating hydride transfer reactions [37]. Fgd2 does not oxidize G6P and its substrate remains unknown [32], [38]. *M. smegmatis* expresses Fgd1 and it lacks Fgd2, whereas both *Mtb* and BCG carry Fgd1 and Fgd2 ([31], [32]; NCBI Accession Number: NC_008596). Both Fgd1 and Fgd2 are also homologs of F_420_-dependent methylenetetrahydromethanopterin reductases (Mer) that are found in methanogenic archaea [34]. To obtain some clues to the nature of the substrate that Fgd2 or Rv0132c acts on, we analyzed the primary structure of this protein based on X-ray crystallographic structures of three well characterized Mer homologs: Fgd1 of *Mtb* (PDB ID: 3B4Y) [32], F_420_-dependent methylenetetrahydromethanopterin reductase from *Methanopyrus kandleri* (MkMer; PDB ID: 1EZW) [39], and an F_420_-dependent secondary alcohol dehydrogenase (Adf; PDB ID: 1RHC) from *Methanoculleus thermophilicus* [40]; *M. kandleri* and *M. thermophilicus* are methanogenic archaea. In Fgd1, His^40^, Ser^73^, Val^74^ and Glu^109^ help to bind F_420_ and these residues, except Ser, are functionally conserved in MkMer and Adf [39], [40] as well as in Rv0132c (Fig. 2). Ser^73^ of Fgd 1 interacts with F_420_ via the oxygen atom of the backbone carbonyl [32], and in Rv0132c and MkMer this residue has been substituted with Gly and in Adf the equivalent position is occupied by a Cys (Fig. 2). Ser, Cys and Gly are highly compatible in terms of their hydrophobicities and sizes [41]–[43]. Hydrophobe compatibility indices for Ser-Cys and Ser-Gly pairs in a scale 1–20 (1 and 20 being least and fully compatible, respectively) are 17.7 and 16.8, respectively [41]. The isoelectric points of Ser, Cys and Gly are 5.7, 6.0 and 5.1, respectively [41]. The volumes of Ser, Cys and Gly are 73, 86 and 48 cubic Angstroms, respectively, which are considered similar [44]; the amino acid volumes range from 48 cubic Angstroms for Gly to 163 cubic Angstroms for Trp. Consequently, the above-mentioned substitutions at Ser^73^ position will not appreciably change the ability of an Fgd1 homolog to bind F_420_. Therefore, in our investigation, we had considered Rv0132c as a potentially F_420_-dependent enzyme. A recent report shows that Rv0132c indeed binds F_420_ [38].

In our next analysis we tried to understand whether Rv0132c has the potential to transform hydrophobic substrates such as mycolic acids. Adf and MkMer interact with hydrophobic substrates whereas Fgd1 accommodates charged glucose-6-phosphate. In Adf the Val^193^ and Leu^227^, which are hydrophobic, not only interact with the hydroxybenzyl unit of F_420_ but also help to position the hydrocarbon chain of the substrate [40]. Similarly, Ala^197^ and Tyr^229^ in MkMer interact with both the F_420_ and the hydrophobic pterin ring of tetrahydromethanopterin [40]. Rv0132c shows partial conservation of these characteristics, as homologous residues in this protein are Ala and Glu, respectively (Fig. 2). In contrast, the equivalent positions in Fgd1 are occupied by Ser and Glu [32], which are less hydrophobic and polar, respectively. In Fgd1, Lys^232^, which has a charged side chain, helps to bind a citrate ion, which is a competitive inhibitor of the enzyme [32]. In Rv0132c, Adf and MkMer, this residue has been replaced with Phe, Trp and Cys, respectively (Fig. 2). Additionally, Fgd1 utilizes a positively charged pocket formed by Lys^198^, Lys^259^, and Arg^283^ to hold the phosphate group of glucose-6-phosphate [32] and these residues are not conserved in Rv0132c (Fig. 2). Hence, Rv0132c has the potential of interacting with a hydrophobic substrate.

We had observed that two tandem Arg residues in the NH_2_-terminus (amino acid residues 1–27, MTGIS **RR**TFGLAAGFGAIGAGGLGGG**C**; bold and underlined, characteristic residues) form a signature for translocation into the periplasmic space via a Tat-dependent protein export pathway which exists in the mycobacteria [45] and the features shown underlined (see above) represent a putative prokaryotic membrane lipoprotein lipid attachment site (PS00013) where Cys^27^ could carry lipophilic substrates [46]. Indeed, as our work was complete, Rv0132c was found to be exported to the cell envelope of *Mtb* [38]. Thus, it is reasonable to assume that Rv0132c could interact with the hydrocarbon chains of the mycolic acids (R group, Fig. 1). The relevance of the demonstrated cellular location of Rv0132c to our findings has been discussed below.

#### Experimental elucidation of the function of Rv0132c

**Figure 3:**
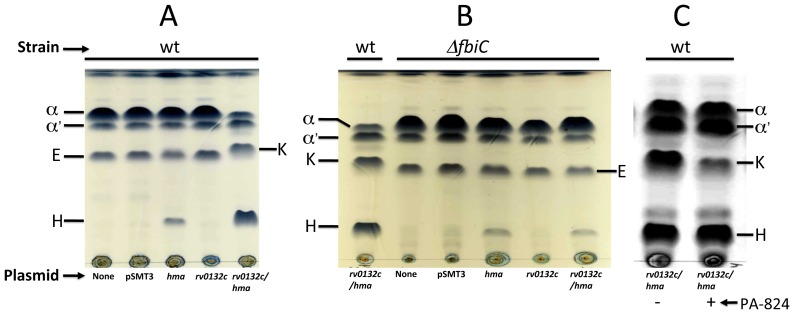
Thin layer chromatography (TLC) profiles of methyl esters of mycolic acids extracted from various *Mycobacterium smegmatis* strains grown in the absence and presence of PA-824. Wild type (wt) and *ΔfbiC* strains of *M. smegmatis* carrying the indicated plasmids were analyzed (lane label, name of plasmid): None, no plasmid; pSMT3, pSMT3 (vector control); *hma*, pEP-hma; *rv0132c*, pEP-rv0132c; *rv0132c/hma*, pEP-rv0132c/hma. (+) and (-), cultivation of *M. smegmatis* (pEP-rv0132c/hma) with and without PA-824 (100 microgram per ml), respectively. Mycolic acid types: α, α′, epoxy (E), hydroxy (H), and keto (K) [Fig. S1 shows the respective chemical structures.]. Panel A: *rv0132c* causing the conversion of H-MAs to K-MAs in wild-type *M. smegmatis*; Panel B: Requirement of *fbiC* for the production of K-MAs in *M. smegmatis* (pEP-rv0132c/hma) [Note: *The left most lane is for wt strain, used as control*]. Panel C: Inhibition of the production of K-MAs in *M. smegmatis* (pEP-rv0132c/hma) by PA-824.

We have tested whether Rv0132c represents an F_420_-dependent hydroxymycolic acids dehydrogenase (fHMAD) by introducing this gene and *hma* into *M. smegmatis*. As expected, the plasmid pEP-hma, which was constructed based on the *E. coli*-mycobacterium shuttle vector pSMT3 [47] and carried *Mtb hma* gene (*rv*0642c) under the control of its native promoter element, produced H-MAs in *M. smegmatis* mc^2^155 or wild-type (wt) strain (Fig. 3A, lane *hma*). The expression of both *hma* and *rv0132c* genes from pEP- rv0132c/hma led to the production of K-MAs (Fig. 3A, lane rv0132c/hma); *rv0132c* alone (pEP- rv0132c) did not provide either H-MAs or K-MAs (Fig. 3A, lane rv0132c). *M. smegmatis*, as such (host control; Fig. 3A, lane None) or while carrying pSMT3 (vector control; Fig. 3A, lane pSMT3), also did not produce either H-MAs or K-MAs; they contained α-, α′-, and E-MAs.

**Figure 4:**
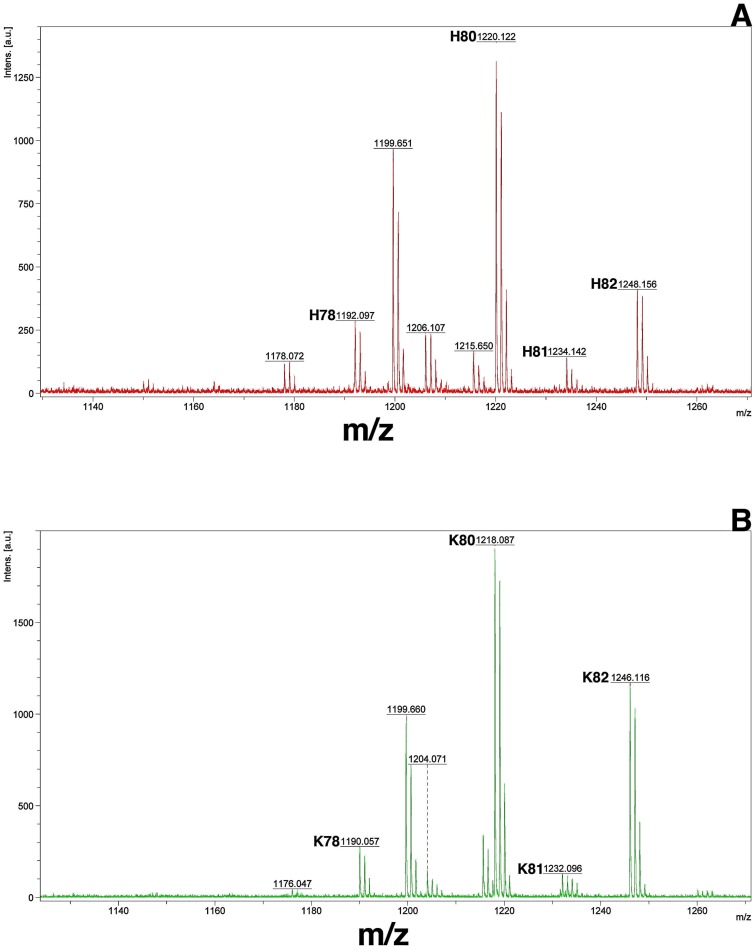
MALDI-TOF mass spectra of methyl esters of hydroxymyoclic acids (A) and ketomycolic acids (B) recovered from engineered *Mycobacterium smegmatis* strains. Hydroxymycolic acids were obtained from the lane *hma* and ketomycolic acids were from lane *rv0132c/hma* (Fig. 3A). Only a part of each spectrum is shown and the annotations for the ion masses are based on reference [23]: labels H & K, ions from hydroxy- and ketomycolic acids preparations; numbers 77–82: total number of carbon atoms in free acids. The unlabeled peaks belong to unidentified species that were present in both preparations.

**Figure 5:**
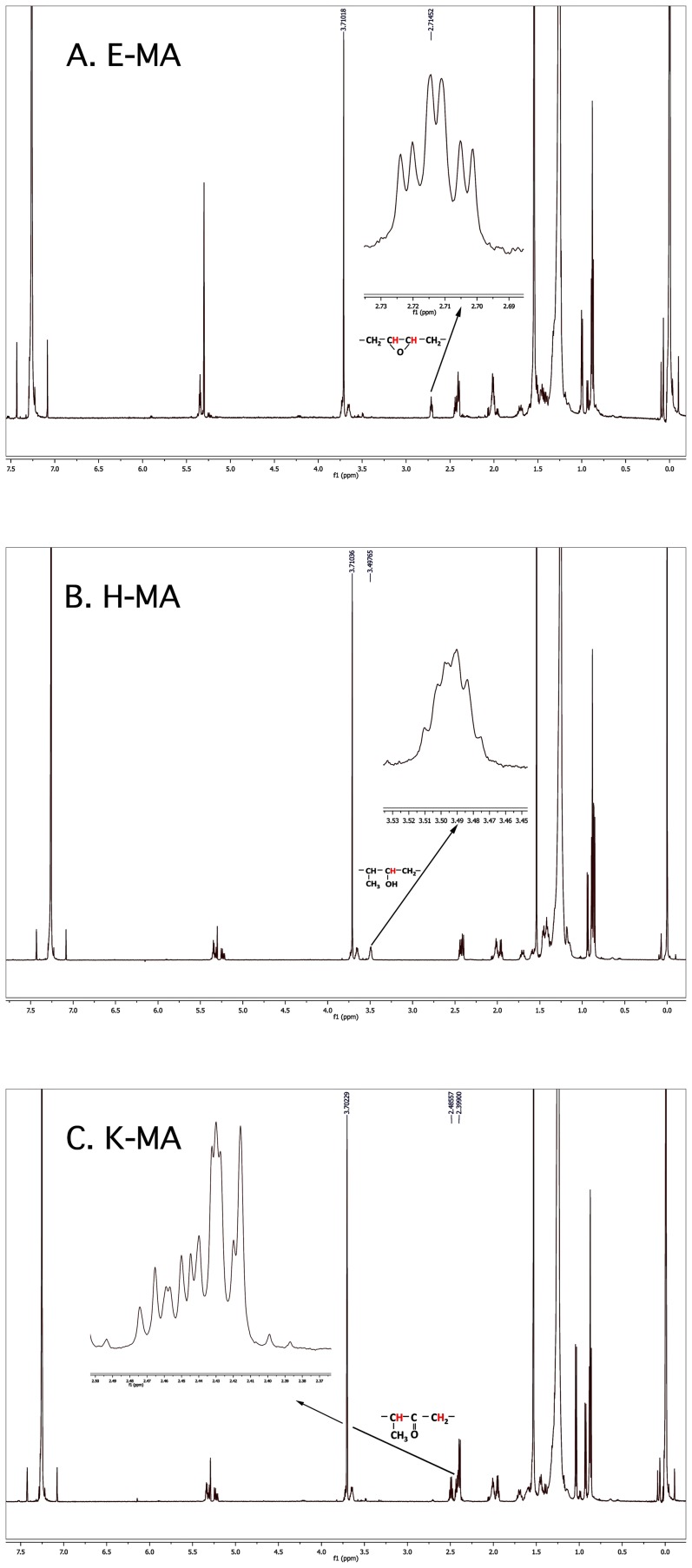
Proton NMR spectra of methyl esters of mycolic acids recovered from engineered *Mycobacterium smegmatis* strains. The sources of hydroxymycolic acids or H-MAs (A) and Ketomycolic acids or K-MAs (B) were same as that indicated in the legend of Fig. 4. Epoxymycolic acids or E-MAs (C) were from lane “None” in Fig. 3. In each case the inset shows expansion of the relevant regions.

The initial identification of the individual mycolic acid bands on the TLC plates was performed via comparison with previously reported patterns [18], [23], [24]. Then we carried out mass spectrometric and NMR spectroscopic analysis with materials recovered from the relevant TLC bands. For the H-MAs and K-MAs bands, MALDI-TOF mass spectrometry yielded spectra that were characteristics of respective myoclic acids with 77–82 carbon atoms (Fig. 4) [23]. The mass for every characteristic H-MA ion (Fig. 4A) was 2 units higher than that for a K-MA (Fig. 4B) and this is consistent with the respective structures shown in Fig. 1. ^1^H NMR data provided more detailed characterization of relevant mycolic acid species and we discuss the findings below with a focus on the H-atoms marked in red in Fig. 1. This analysis is based on previously reported NMR data on mycolic acids [23], [27], [29], [48], [49]. The resonances at 2.7 ppm observed with the E-MA preparation obtained from *M. smegmatis* mc^2^155 cells (Fig. 5A) were characteristics of the methine protons associated with a *trans*-epoxide group [23]. In the spectrum obtained with the H-MAs preparation from *M. smegmatis* (pEP-hma) strain (Fig. 5B) the resonances for the above-mentioned epoxy group were not seen and instead it exhibited a resonance at 3.5 ppm representing the methine proton on the carbon that carried the characteristic hydroxyl group of H-MA. Similarly, the ^1^H resonances of the methylenic and methine groups that flank the carbonyl group in K-MAs were found at 2.31–2.39 ppm in the spectra for the K-MA preparation from *M. smegmatis* (pEP-rv0132c/hma) strain (Fig. 5C). The spectra for the E-MA, H-MA and K-MA preparations exhibited the following common resonances and this observation is consistent with previous reports [23], [27], [29], [48], [49] (Fig. 5A–C): 1.29 ppm – broad, isolated methylene proton; 0.85 ppm - triplet, terminal methyl groups; 3.71 ppm – singlet, methyl ester; 2.50 ppm - multiplet, methine at postion C-2 with respect to the terminal carboxyl group (see within the box at the left corner of Fig. 1). None of the above spectra showed the resonances of the protons that are associated with the cyclopropane groups of mycolic acids produced by *Mtb*; these resonances appear at −0.40, 0.50, and 0.58 ppm for cis-cyclopropanation and 0.01–0.16 ppm for trans-cyclopropanation [49]. Major mycolic acids produced by *M. smegmatis* lack cyclopropanation under normal growth conditions [23], [50]. This modification occurs during growth at 25°C [51] and the growth temperature in our study was 37°C.

The above-described analysis showed that the heterologous expression of *Mtb hma* in *M. smegmatis* caused the suppression of the synthesis of E-MAs and the production of H-MAs, and Rv0132c protein converted the H-MAs to K-MAs. Thus, in *Mtb* Rv0132c encoded a hydroxymycolic acid dehydrogenase (HMAD).

The next step was to determine if HMAD was coenzyme F_420_-dependent. The *fbiC* is a key gene for the production of F_420_ chromophore in mycobacteria [52] and mycobacterial strains lacking a functional *fbiC* gene are devoid of this coenzyme [52], [53]. We found that a *M. smegmatis ΔfbiC* strain [53] generated H-MAs but not K-MAs when complemented with pEP-rv0132c/hma (Fig. 3B, right most lane or the lane rv0132c/hma for *ΔfbiC*). Complementation with pEP-hma also provided H-MAs in *M. smegmatis ΔfbiC* (Fig. 3B, lane *hma*) and pEP-rv0132c did not provide either K-MAs or H-MAs (Fig. 3B, lanes rv0132c); the left most lane (lane rv0132c/hma for wild-type (wt) strain) served as a positive control, where production of K-MAs was observed. Hence, HMAD required F_420_ for activity and we call it hereafter fHMAD.

In this context we address two sets of contradicting reports in the literature that concern the biosynthesis of H-MAs and K-MAs in *M. smegmatis* strains carrying clones for the *Mtb hma* gene. In one case the *hma* gene caused the synthesis of both H-MAs and K-MAs [23], [24] and in the latter only H-MAs were found in the recombinant [18]. Our result is consistent with the latter [18], as the conversion of a hydroxyl group to a keto group would be catalyzed by an electron transfer enzyme or dehydrogenase such as fHMAD, and not by a methylase/hydrase activity such as seen in Hma.

Fig. 1 shows two mycobacterial MA oxygenation pathways, one of which leads to H-MAs, K-MAs and M-MAs, and the other is for the production of E-MAs. It has been shown that when the former operates, the latter is suppressed [17], [23], [24], [27]. We observed a more stringent form of this regulation in our studies. The data in Fig. 3A show that when *M. smegmatis* was made capable of producing K-MAs, it did not produce E-MAs; a comparison of rv0132c/hma lane with any other lane in Fig. 3A leads to this conclusion. This effect was not due to the Rv0132c protein or the DNA elements cloned into pEP-rv0132c/hma, as their presence did not suppress E-MA production when the host lacked *fbiC* (Fig. 3B, lanes hma, rv0132c and rv0132c/hma). Hence it could be hypothesized that K-MAs either inhibit one or more E-MA synthesis enzymes and/or suppresses the expression of respective genes. Other possibilities are the interference with the translocation of the precursor of E-MAs to the modification site such as periplasm or a flux-based competition between the two pathways. We also observed that the cellular level of H-MAs increased when K-MAs were produced (Fig. 3A, lane pEP-rv0132c/hma). It is possible that K-MAs enhanced the activities of one or more enzymes that generate H-MAs from α-mycolic acids (Fig. 1) and/or increased the expressions of their genes, and the prevailing fHMAD activity was not at par with the rate of H-MA production. The other explanation is that in *M. smegmatis* (pEP-rv0132c/hma) the cellular level of Hma activity was much higher than that of fHMAD. In this context we note that the overproduction of M-MAs through over-expression of Mma3 or CmaB suppresses K-MAs in *Mtb* [18], [22]. A more detailed study is needed to elucidate the mechanisms underlying these competitions between mycolic acids oxygenation pathways.

### Inhibition of fHMAD by PA-824

PA-824, a new TB drug, inhibits the formation of K-MAs and causes an accumulation of H-MAs in *Mtb* [54]. We tested whether this effect is specifically due to the inhibition of fHMAD. As shown in Fig. 3C, in the presence of PA-824, *M. smegmatis* (pEP-rv0132c/hma) accumulated a high level of H-MAs and contained a reduced level of K-MAs (Fig. 3C). Hence, PA-824 inhibited the heterologously expressed fHMAD. To establish further that fHAMD was inhibited by PA-824, we determined the relative levels of K-MAs in *M. smegmatis* (pEP-rv0132c/hma) cultivated in the presence of this drug at various concentrations. The results showed that the inhibition began at a PA-824 concentration between 10–25 microgram per ml culture and increased further as the drug concentration was raised (Fig. S2). Such a dose-dependent increase in the inhibition of K-MA synthesis by PA-824 has been reported also for *M. tuberculosis* [54]. However, the K-MA synthesis process in *M. smegmatis* (pEP-rv0132c/hma) was much less sensitive to PA-824 than that observed in wild-type *M. tuberculosis*; in *M. tuberculosis* this inhibition begins at a PA-824 concentration between 30-60 nanogram per ml culture [54]. It is possible that the higher minimum inhibitory concentration of PA-824 observed with *M. smegmatis* (pEP-rv0132c/hma) was due to the presence of a higher level of Rv0132c protein in this recombinant strain; *rv0132c* was expressed from a multi-copy plasmid [47] and was driven by both the native promoter as well as the strong and constitutive mycobacterial *hsp60* promoter [47], [55]. Another explanation is that compared to *M. tuberculosis*, *M. smegmatis* takes up PA-824 poorly and as a result for achieving an inhibitory concentration of the drug inside the cell, it had to be supplied in the culture medium at a higher concentration; wild-type *M. smegmatis* is naturally resistant to PA-824 [56]. Nevertheless, the results presented in Figs. 3C and S2 show that the phenomenon of inhibition of K-MAs synthesis by PA-824 that was observed in wild-type *M. tuberculosis* could be reproduced in a *M. smegmatis* strain carrying cloned *hma* and *rv0132c* genes from the former.

PA-824 kills *Mtb* under both aerobic and anaerobic conditions [54], [57], [58]. The anaerobic killing occurs through the reduction of PA-824 by an F_420_H_2_-dependent nitroreductase called Ddn, which is followed by the production of toxic NO [57]. Since F_420_H_2_ is produced by Fgd1, *Mtb* and BCG strains lacking Fgd1 activity are resistant to PA-824 [54]. The aerobic killing of *Mtb* by PA-824 has been thought to occur due to the elimination of K-MAs via unknown mechanisms [54], [58]. Our data has now linked this concept to a gene, *rv0132c*. Curiously, F_420_ is an integral part of both mycobacterial systems, Ddn and fHAMD, that interact with PA-824.

As mentioned above, fHMAD found to be exported to the cell envelope of *Mtb* [38]. Also, it is thought that complete mycolic acids are transported to the plasma membrane as trehalose monomycolates or TMM [59]. In combination these observations suggest that in *Mtb* at least one additional modification, formation of keto group, of otherwise complete mycolic acids occur within the cell envelop.

### Conclusion

The hydroxymycolic acid dehydrogenase of *Mtb* was shown to be an F_420_-dependent enzyme encoded by the ORF Rv0132c and it is inhibited by PA-824, a new TB drug.

Our data suggest that there is only one *bona fide* Fgd in the mycobacteria. Citing the lack of glucose-6-phosphate dehydrogenase activity in Rv0132c, it has been recently suggested that this protein should no longer be called Fgd2, and Fgd1 should be called simply Fgd [38]. Our data supports this proposal and provides a functional name for Rv0132c, F_420_-dependent hydroxymycolic acid dehydrogenase (fHMAD). Coenzyme F_420_ is universally present and essential in the strictly anaerobic methanogenic archaea [37]. In the bacterial domain, a similarly wide distribution of this deazaflavin derivative is seen in the Actinobacteria phylum, which includes the mycobacteria [35], [36]. Every mycobacteria examined thus far contains F_420_ [35], [36]. As mentioned above, in the hydride transfer function F_420_ mimics NAD(P). The mid-point electrode potential of the F_420_/F_420_H_2_ couple −360 mV, which is 40 mV lower than that of the nicotinamides [37]. Perhaps in the mycobacteria F_420_ participates in a set of hydride transfer reactions that cannot be accomplished at all or efficiently by the nicotinamides due to thermodynamic reasons, such as a need to operate at a lower redox potential. Such a specialized role has now been seen in the neutralization of nitrosative stress [53] (via a chemical reaction with Fgd-derived F_420_H_2_) and in the introduction of a key functionality to the complex mycobacterial cell envelope (the fHMAD reaction as demonstrated here). Both of these actions bring resilience to the mycobacteria against environmental stresses such as those imposed by the human immune system. It is noteworthy, that the current report presents the first example for the involvement of F_420_ in the biosynthesis of mycobacterial cell wall. The nitroreductase (Ddn) that helps to activate PA-824 with F_420_H_2_ and the F_420_-dependent enzymes that allow the mycobacteria to decolorize triphenylmethane dyes or to degrade aflatoxins [57], [60]–[62] could also fulfill yet to be described key and normal physiologically relevant cellular functions in these organisms.

## Materials and Methods

### Oligonucleotides, Plasmid, DNA, Bacteria and Growth Conditions

Oligonucleotides, plasmids and bacteria used in this study have been described in the Supporting Material (Table S1). *M. tuberculosis* H37Rv chromosomal DNA was obtained from the National Institutes of Health's TB Vaccine Testing and Research Materials Contract (TBVTRMC) at the Colorado State University. *E. coli* was grown in Luria-Bertani broth or solid media. *Mycobacterium smegmatis* strains were grown in Middlebrook 7H9 broth or on agar solidified medium with 0.2% glycerol as the carbon and energy source [63]. For liquid cultures Tween 80 at the concentration of 0.05% was also added. When required, *M. smegmatis* strains bearing antibiotic resistance genes were selected on or grown with kanamycin and hygromycin at the concentration of 20 and 150 microgram/ml, respectively, and for similar work with *E. coli* strains ampicillin, kanamycin, and hygromycin concentrations were 100, 20, and 150 microgram/ml, respectively. To study the effect of PA-824 on the mycolic acids content of *M. smegmatis* (pEP-rv0132c/hma), a freshly inoculated culture was grown overnight to an optical density of 0.3 at 600 nm (as measured by use of a DU800 UV/Vis Spectrophotometer, Beckman Coulter, Brea, CA). It was then supplemented with PA-824 to a desired final concentration from a stock solution (80 mg/ml) in DMSO and grown for additional 36 h. The control culture received DMSO at a concentration of 1.25 ml/liter. PA-824 was a gift from Global Alliance for TB Drug Development (New York, NY) through the Global Health program of the RTI International (Research Triangle Park, NC).

### Molecular Biology Techniques

*M. smegmatis* chromosomal DNA was isolated as described previously [63]. Transformation of *M. smegmatis* with plasmids was performed via electroporation [64] at 2.5 KV using an Electroporator 2510 (Eppendorf North America, Hauppauge, NY) and a cuvette with a 0.2 cm electrode-gap. For PCR amplification, Phusion polymerase with the GC buffer (Finnzymes Inc., Woburn, MA) was used. Plasmid purification and DNA recovery from agarose gels were done using Qiaprep and Qiaquick columns (Qiagen Inc., Valencia *CA*), respectively. Manipulations of DNA were performed using standard methods [65].

### Construction of Protein Expression Plasmids and Bacterial Strains

The protein expression plasmids were based on pSMT3, a mycobacteria–*Escherichia coli* shuttle vector that allows selection for hygromycin resistance and gene expression under the control of the strong and constitutive *hsp60* promoter [47], [55]. To generate the plasmids pEP-hma and pEP-rv0132c for the expression of *hma* (*rv*0642c) and *rv0132c* of *M. tuberculosis*, respectively, the corresponding coding sequences along with the respective upstream regions bearing the promoters and ribosome-binding sites (253 bp for *hma* and 316 bp for *rv0132c*) and a bit of the downstream sequences (4 bp for *hma* and 20 bp for *rv0132c*) were PCR-amplified from *M. tuberculosis* H37Rv chromosomal DNA and cloned into pSMT3; the cloning sites were EcoRV and ClaI for *hma* and BamHI and EcoRV for *rv0132c*. The primers used for this work have been described in the Supporting Material (Table S1). The cloned genes in pEP-hma and pEP-rv0132c were expressed in *M. smegmatis* from their native promoters and perhaps also from the plasmid resident mycobacterial *hsp60* promoter. The plasmid pEP-rv0132c/hma that allowed simultaneous expression of *hma* and *rv0132c* was constructed by cloning the *hma* coding sequence along with the respective upstream and downstream sequences as mentioned above at the EcoRV and ClaI sites (or at the 3′end of the *rv0132c* segment) of pEP-rv0132c. The construction of *M. smegmatis ΔfbiC::aph* strain has been described previously [53].

### Preparation and Analysis of Mycolic Acid Methyl Esters

Mycolic acid methyl esters (MAMEs) were prepared as described previously [66]. Briefly, pelleted mycobacterial cells were saponified via incubation in 15% tetrabutyl ammonium hydroxide at 110°C overnight, followed by the addition of water, diazomethane, and dichloromethane and shaking at room temperature. From this mixture the MAMEs were recovered in the dichloromethane fraction and washed sequentially with equal volumes of water, 0.1 N HCl and water. The dichloromethane solution of MAMEs was dried under a stream of nitrogen, dissolved in a toluene-acetonitrile mixture (2:1), and then precipitated at room temperature with an addition of acetonitrile (final toluene:acetonitrile, 2∶3). The pellet of MAMEs was dissolved in dichloromethane. Analysis of MAMEs was carried out by thin layer chromatography (TLC) on an aluminum-backed silica gel plate (10×10 cm, Merck 5735-silica gel 60F254) by multiple developments using a solvent comprised of petroleum ether and diethyl ether (9∶1). Mycolic acid spots were revealed by charring at 110°C for 15 min after spraying with 5% ethanolic molybdophosphoric acid [66].

### Mass Spectrometric and NMR Analysis of Mycolic Acids

This work concerned the methyl esters of hydroxy- and keto-mycolic acids. After performing multi-lane TLC separation for a sample, a terminal lane was cut off and processed for color development as described above. Then using a relevant band in this lane as a guide, the desired mycolic acid spots (silica layer) were scrapped off from the rest of the lanes. From the recovered silica particles, mycolic acids were extracted with dichloromethane and analyzed via MALDI-TOF mass spectrometry at School of Chemical Sciences Mass Spectrometry Laboratory at the University of Illinois at Urbana-Champaign. The Bruker peptide calibration mixture II (Angiotensin II, Angiotensin I, Substance P, Bombesin, ACTH clip 1–17, ACTH clip 18–39, Somatostatin 28, Bradykinin Fragment 1–7, and Renin Substrate Tetradecapeptide porcine Covered mass range: ∼700 Da – 3200 Da) was used for calibration and the matrix was 2,5-dihydroxybenzoic. A Bruker UltrafleXtreme mass spectrometer (Fahrenheitstr. 4,D-28359 Bremen, Germany) equipped with a smart beam II laser was used in the positive mode to acquire MALDI-TOF mass spectra. Samples were analyzed in the Reflectron mode.

A Bruker Avance III 600 MHz available at the NMR Laboratory, Department of Chemistry, Virginia Tech, was used to obtain ^1^H NMR spectra of the purified mycolic acid methyl esters preparations described above. The solvent was CDCl_3_ (100% D) and the reported chemical shifts were relative to the methyl resonances of tetramethylsilane (0 ppm).

## Supporting Information

Figure S1 Structures of mycolic acids in and *Mycobacterium tuberculosis* complex and *Mycobacterium smegmatis*. The detailed structures of R groups in various mycolic acids are shown [4]. The reference cited here is listed in File S1.
(TIF)

Figure S2 Dose-dependent inhibition of K-MA production in *M. smegmatis* (pEP- rv0132c/hma) by PA-824. Wild-type *M. smegmatis* was used as control; the data in Fig. 3 show that neither the expression constructs pEP-hma and pEP-rv0132c nor the vector pSMT3 allow the production of K-MAs in *M. smegmatis*. The other details of the study have been presented in the MATERIALS AND METHODS. Mycolic acid types: α, α′, epoxy (E), hydroxy (H), and keto (K) [Fig. 1S shows the respective chemical structures.].
(TIF)

Table S1 Oligonucleotides, plasmids, and strains. The references cited in this table are listed in File S1.
(DOC)

File S1 References for Table S1 and Figure S1.
(DOC)
