# Supplementary figures and images for: Rv0132c of Mycobacterium tuberculosis Encodes a Coenzyme F420-Dependent Hydroxymycolic Acid Dehydrogenase
Source: PLoS One. 2013 Dec 11;8(12):e81985. doi: 10.1371/journal.pone.0081985 (PMC3859598; doi:10.1371/journal.pone.0081985)

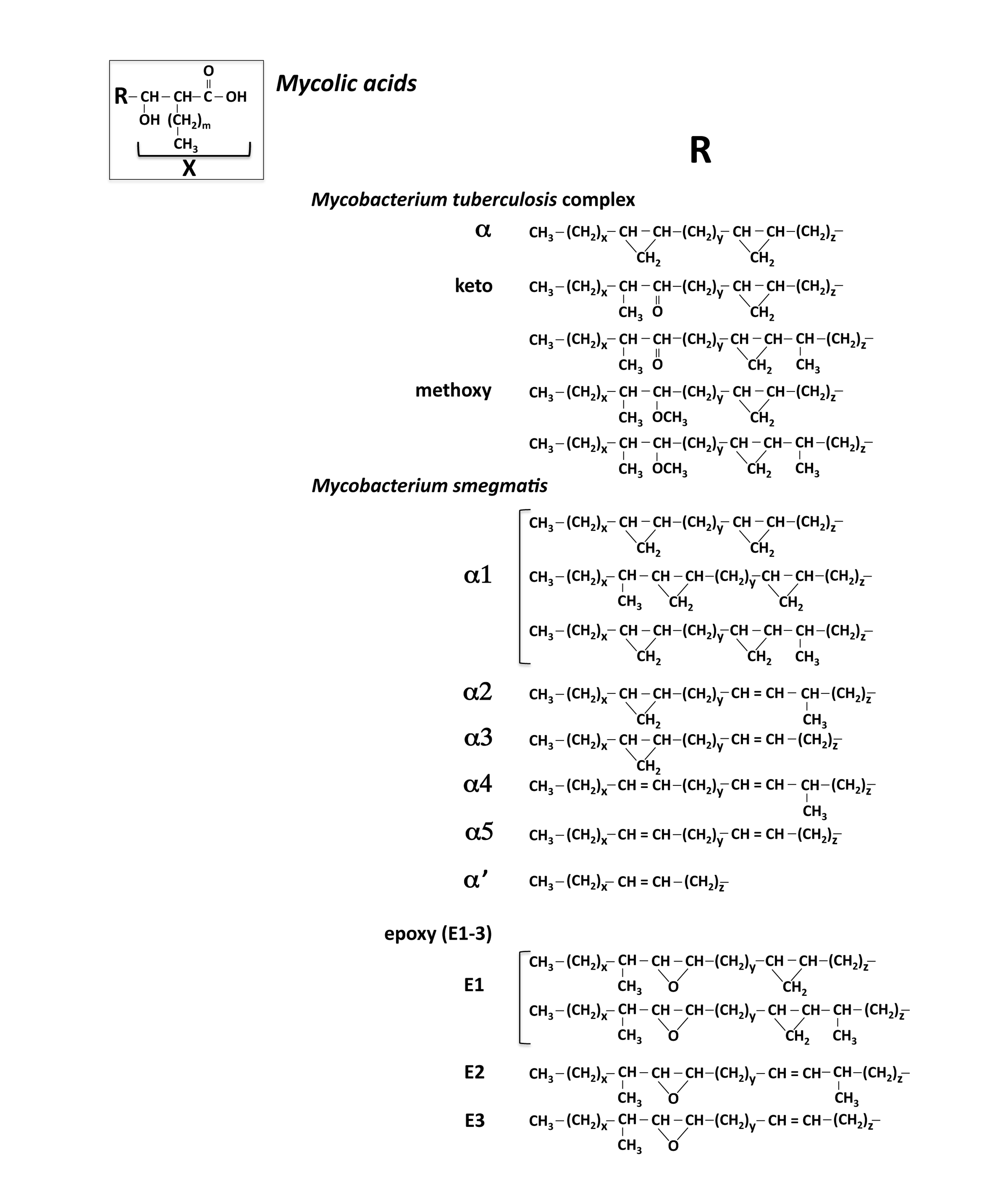

Supplement: Figure S1 — Structures of mycolic acids in and Mycobacterium tuberculosis complex and Mycobacterium smegmatis. The detailed structures of R groups in various mycolic acids are shown [4]. The reference cited here is listed in File S1. (TIF) [file pone.0081985.s001.tif]

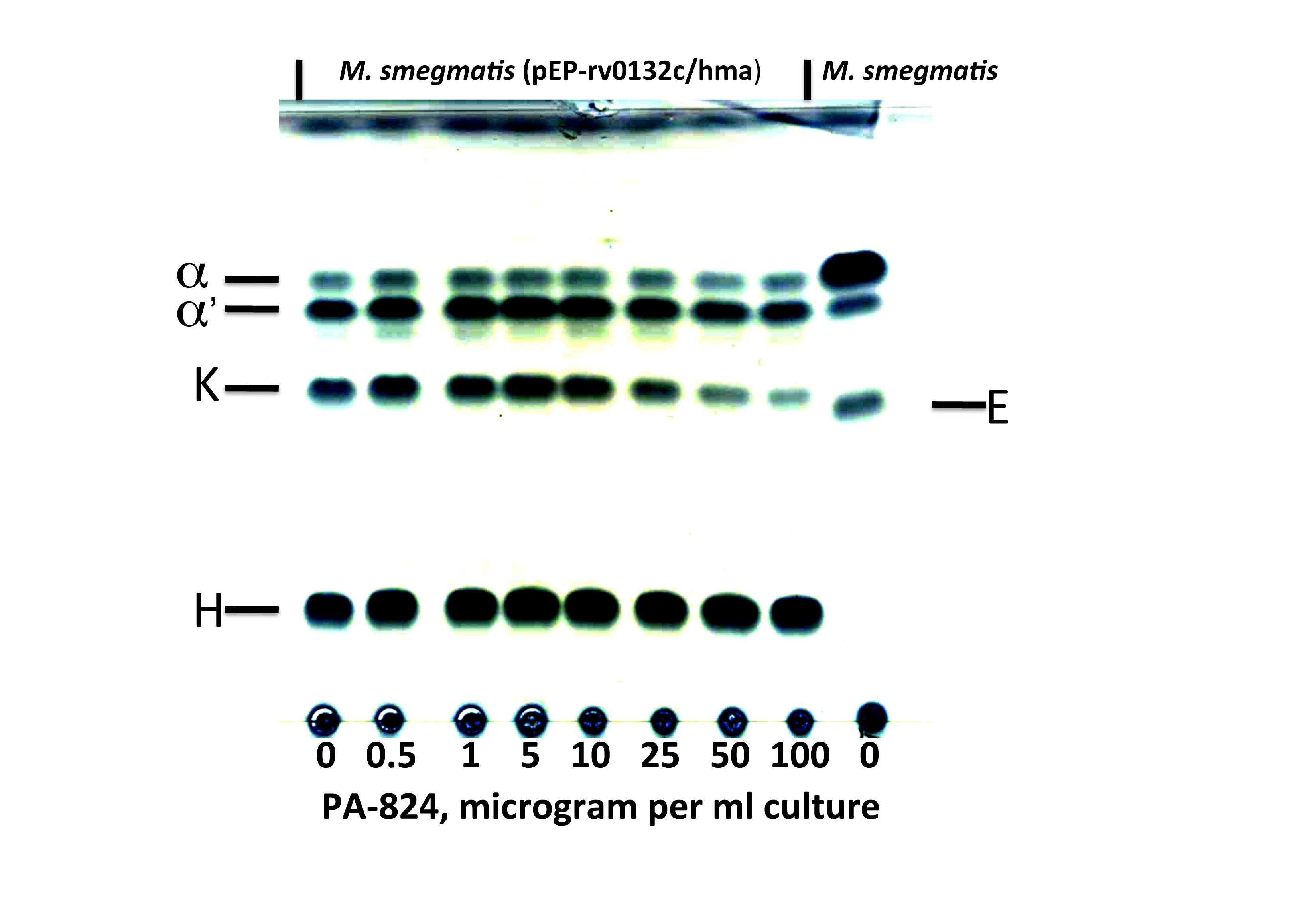

Supplement: Figure S2 — Dose-dependent inhibition of K-MA production in M. smegmatis (pEP- rv0132c/hma) by PA-824. Wild-type M. smegmatis was used as control; the data in Fig. 3 show that neither the expression constructs pEP-hma and pEP-rv0132c nor the vector pSMT3 allow the production of K-MAs in M. smegmatis. The other details of the study have been presented in the MATERIALS AND METHODS. Mycolic acid types: α, α′, epoxy (E), hydroxy (H), and keto (K) [Fig. 1S shows the respective chemical structures.]. (TIF) [file pone.0081985.s002.tif]
