# Supplementary material for: Rv0132c of Mycobacterium tuberculosis Encodes a Coenzyme F420-Dependent Hydroxymycolic Acid Dehydrogenase
Source: PLoS One. 2013 Dec 11;8(12):e81985. doi: 10.1371/journal.pone.0081985 (PMC3859598; doi:10.1371/journal.pone.0081985)
Supplement: Table S1 — Oligonucleotides, plasmids, and strains. The references cited in this table are listed in File S1. (DOC) [file pone.0081985.s003.doc]

**Table S1. Oligonucleotides, plasmids, and strains**

| **Item** | | **Reference (Source)** |
| --- | --- | --- |
| **Oligonucleotides for generating amplicons for cloning into pSMT3** | | |
| Name (gene amplified) | Sequence (underlined engineered restriction site) | This work |
| Hma_1F (*hma* or *rv0642*) | 5’-ccattaccgatatccagtccctgcaaccgc3’ (EcoRV) | This work |
| Hma_2R (*hma* or *rv0642*) | 5’-tctgatcgatgggcttaggccgcggcacc-3’ (ClaI) | This work |
| Rv0132c _1F (*rv0132c*) | 5’-gggatcccacagcgccgccc-3’ (BamHI) | This work |
| Rv0132c _2R (*rv0132c*) | 5’-ggatatcgtaggtgcggtctagc-3’ (EcoRV) | This work |
| **Plasmids** | | |
| Name | Details |  |
| pSMT3 | Mycobacteria - E. coli shuttle vector with hygromycin resistance gene and hsp60 promoter for use in mycobacteria |  |
| pEP-hma | pSMT3 + *M. tuberculosis* *hma* gene with 253 bp upstream and 4 bp downstream as EcoRV-ClaI fragment | This work |
| pEP-rv0132c | pSMT3 + *M. tuberculosis rv0132c* gene with 316 bp upstream and 20 bp downstream as BamHI-EcoRV fragment | This work |
| pEP-rv0132c /hma | pEP-*rv0132c* + *M. tuberculosis hma* gene with 253 bp upstream and 4 bp downstream as EcoRV-ClaI fragment | This work |
| **Bacterial strains** | | |
| Name | Genotypes and other details |  |
| *E. coli* TOP10 (plasmid construction host) | F- *mcr*A Δ(*mrr-hsd*RMS*-mcr*BC) φ80*lac*ZΔM15 Δ*lac*X74 *nup*G *rec*A1 *ara*D139 Δ(*ara-leu*)7697 *gal*E15 *gal*K16 *rps*L(Str^R^) *end*A1 λ^-^ | (Life Technologies Corp., Carlsbad, CA) |
| *Mycobacterium smegmatis* mc^2^155 | Easily transformable *M. smegmatis* (parent strain) | This work |
| *M. smegmatis* (pEP-hma) | *M. smegmatis* mc^2^155 carrying pEP-hma | This work |
| *M. smegmatis* (pEP- rv0132c) | *M. smegmatis* mc^2^155 carrying pEP-*rv0132c* | This work |
| *M. smegmatis* (pEP- rv0132c /hma) | *M. smegmatis* mc^2^155 carrying pEP-*rv0132c* /hma | This work |
| *M. smegmatis* ΔfbiC::aph | *fbiC* coding sequence replaced with an *aph* cassette |  |
