# Supplementary material for: Rv0132c of Mycobacterium tuberculosis Encodes a Coenzyme F420-Dependent Hydroxymycolic Acid Dehydrogenase
Source: PLoS One. 2013 Dec 11;8(12):e81985. doi: 10.1371/journal.pone.0081985 (PMC3859598; doi:10.1371/journal.pone.0081985)
Supplement: File S1 — References for Table S1 and Figure S1. (DOC) [file pone.0081985.s004.doc]

**References for Table S1 and Figure S1**

1. Garbe TR, Barathi J, Barnini S, Zhang Y, Abou-Zeid C, et al. (1994) Transformation of mycobacterial species using hygromycin resistance as selectable marker. Microbiology 140 ( Pt 1): 133-138.

2. Snapper SB, Melton RE, Mustafa S, Kieser T, Jacobs WR, Jr. (1990) Isolation and characterization of efficient plasmid transformation mutants of *Mycobacterium smegmatis*. Mol Microbiol 4: 1911-1919.

3. Purwantini E, Mukhopadhyay B (2009) Conversion of NO_2_ to NO by reduced coenzyme F_420_ protects mycobacteria from nitrosative damage. Proc Natl Acad Sci U S A 106: 6333-6338.

4. Laval F, Haites R, Movahedzadeh F, Lemassu A, Wong CY, et al. (2008) Investigating the function of the putative mycolic acid methyltransferase UmaA: divergence between the *Mycobacterium smegmatis* and *Mycobacterium tuberculosis* proteins. J Biol Chem 283: 1419-1427.
